# Experiences and preferences for advance care planning following a diagnosis of dementia: Findings from a cross-sectional survey of carers

**DOI:** 10.1371/journal.pone.0286261

**Published:** 2023-06-12

**Authors:** Jamie Bryant, Elise Mansfield, Emilie Cameron, Rob Sanson-Fisher

**Affiliations:** 1 School of Medicine and Public Health, College of Health, Medicine and Wellbeing, University of Newcastle, Callaghan, Australia; 2 Equity in Health and Wellbeing Program, Hunter Medical Research Institute, New Lambton Heights, Australia; PLOS ONE, UNITED KINGDOM

## Abstract

**Background:**

Future medical and financial planning is important for persons with dementia given the impact of the disease on capacity for decision making.

**Aims:**

To explore from the perspective of carers of persons with dementia: (1) Participation in future medical and financial planning by the person they care for, including when planning was undertaken and the characteristics associated with having an advance care directive completed; (2) The type of healthcare providers who discussed advance care planning following diagnosis; and (3) Preferences for timing of discussions about advance care planning following diagnosis.

**Methods:**

Recruitment and data collection took place between July 2018 and June 2020. Carers of persons with dementia aged 18 years and older were mailed a survey. Participants completed questions regarding completion of various future planning documents by the person they support, including time of completion and who discussed advance care planning following diagnosis. Participants were presented with information about the benefits and consequences of early and late discussions of advance care planning and asked when discussions about advance care planning were best initiated.

**Results:**

198 carers participated. Most participants were female (74%) and had been a carer for more than 2 years (82%). Most participants reported that the person with dementia they support had made a Will (97%) and appointed an Enduring Guardian (93%) and Enduring Power of Attorney (89%). Only 47% had completed an advance care directive. No significant associations were found between characteristics of persons with dementia and completion of an advance care directive. Geriatricians (53%) and GPs (51%) most often discussed advance care planning following diagnosis. Most carers thought that discussions about advance care planning should occur in the first few weeks or months following diagnosis (32%), at the healthcare provider’s discretion (31%), or at the time of diagnosis (25%).

**Conclusions:**

More than half of persons with dementia do not have an advance care directive. There is variability in preferences for timing of discussions following dementia diagnosis.

## Introduction

Advance care planning is defined as a process that “enables individuals who have decisional capacity to identify their values, to reflect upon the meanings and consequences of serious illness scenarios, to define goals and preferences for future medical treatment and care, and to discuss these with family and health-care providers” [1]. Advance care planning provides an opportunity for individuals to indicate, ahead of time, the type of care they want if they become unable to express these preferences themselves. Advance care planning will often lead to the completion of an advance care directive and/or the appointment of a substitute decision maker to help ensure care preferences are respected. Optimally, advance care planning should occur as a series of conversations, and patients’ priorities and preferences should be reviewed and updated over time as needs change [2]. Systematic reviews indicate that engaging in advance care planning has numerous benefits. These include improving the likelihood that care provided aligns with the patients’ goals and values, reducing unwanted and unnecessary admission to hospital at the end of life, improving patient satisfaction and quality of life, and relieving family anxiety and burden of decision making [3–5]. Healthcare savings have also been found for some patient groups including community-dwelling people with dementia and people residing in nursing homes [6, 7]. Within Australia, all Australian states and territories recognise advance care planning under either statutory law or common law, however there are differences between jurisdictions in terminology and requirements for completing and enacting advance care planning processes.

The significance of advance care planning for people with dementia is widely acknowledged [2, 8, 9]. Dementia refers to a collection of neurological disorders that are characterised by progressive memory loss and cognitive impairment [10]. In 2022, it is estimated that there are 487,500 people living with dementia in Australia, with this figure projected to increase to more than 1 million people by 2058 [11]. Ensuring people diagnosed with dementia are supported to engage in advance care planning is critical given the unpredictable progressive nature of the disease, and its impact on decisional capacity [12]. Despite this, there is little data available about participation in advance care planning by people with dementia. A recent medical record audit estimated that only approximately 60% of Australians with dementia have advance care planning documentation located in their medical records [13]. Other studies conducted in the UK have shown that people with dementia are more likely to have undertaken financial planning (e.g. completing a will or appointing an individual to make financial and legal decisions on their behalf) than documented future health care wishes [14].

Initiating and facilitating discussions about advance care planning with patients and their families is a complex task that requires the provision of clear and easily understandable information delivered in a way that is sensitive, compassionate, empathetic and is tailored to the individual needs of each patient [15]. While advance care planning can be initiated at any time in a person’s life, it is likely that a diagnosis of a terminal disease like dementia may prompt discussions. Known barriers to discussing advance care planning include difficulties with defining the right moment and expressed attitudes from some healthcare providers that the patient should initiate advance care planning [16]. Choosing the right time to have discussions with people with dementia can be challenging given the unpredictable duration and trajectory of the illness, and that dementia is often not perceived as a terminal disease [17, 18]. There has been debate among professionals involved in the care of people with dementia about the optimal time to commence advance care planning discussions [19, 20]. Given the progressive nature of the disease, it is too late to commence advance care planning discussions when dementia is significantly progressed and decision-making capacity significantly impacted [20, 21]. While some suggest advance care planning should therefore start as soon as the diagnosis is made when the person is able to be actively involved and patient preferences, values, needs and beliefs can be elicited [19], others consider the point of diagnosis too early, and suggest the right time to raise advance care planning remains unclear [20]. While caregivers of people with dementia generally agree that discussions about advance care planning are both appropriate to have but also difficult [22, 23], considerably less research has examined when persons with dementia and their caregivers think discussions should occur, and work that has been conducted is qualitative. Sussman et al examined perceptions about optimal timing of discussions about future care amongst people with dementia and their caregivers and found variation in perceptions; while some people with dementia were ready to have discussions, many more persons with dementia and their families felt threatened by discussions that necessarily required them to consider future deterioration [23]. As caregivers witness firsthand the impact of cognitive decline and play a significant role in providing day-to-day support and making care decisions for the person they support, they have a unique perspective to offer about the potential benefits and consequences of different timing of advance care planning discussions.

Research has shown that advance care planning conversations are not often initiated by the person living with dementia [24]. Facilitating advance care planning is therefore the responsibility of health care providers involved in a patient’s care [8, 25]. However, there is continued debate about which professional group should take overall responsibility for encouraging engagement with advance care planning among patients, with many healthcare providers reporting that they lack the skills, resources and training to support patients to engage with advance care planning [20]. The process of diagnosis and ongoing management of dementia typically includes multiple healthcare providers, yet it is unclear who is most often involved in advance care planning discussions for people with dementia. It has been suggested that general practitioners have a key role to play in initiating advance care planning with persons with dementia given they often have long lasting relationships and act as gatekeepers in the healthcare system [8, 26]. To date, few studies have explored who initiates advance care planning conversations. This information is critical to understand current practices and preferences, and to guide the provision of advance care planning for people with dementia.

### Aims

This study aimed to explore in a sample of carers of people diagnosed with dementia:

1. The prevalence of participation in future medical planning (Enduring Guardian, advance care directive) compared to financial planning (Will, Enduring Power of Attorney) by the person they care for, including the characteristics associated with having an advance care directive completed.
2. The type of healthcare providers who discussed advance care planning following a diagnosis of dementia.
3. Preferences for timing of discussions about advance care planning following a diagnosis of dementia.

## Materials and methods

### Design

Cross-sectional survey completed by carers of people with dementia living in two states of Australia.

### Participants

Carers aged 18 years and older who were a primary source of support to a person with a confirmed diagnosis of dementia living in the community were eligible to participate. Carers were defined as any individual who self-reported having a significant personal relationship with a person living with dementia and was a main source of emotional and practical support. Carers of persons with dementia who permanently resided in a residential aged care facility were ineligible to participate.

### Recruitment and data collection

Carers were identified from geriatrician clinics, aged care provider records, respite centres and carer support groups and mailed a study information pack. This included a cover letter, a detailed information statement, a hard copy of the survey and a reply-paid envelope. Individuals who consented to participating were asked to return their completed survey using the supplied reply-paid envelope. Recruitment and data collection took place between July 2018 and June 2020.

### Measures

#### Participation in advance care planning

Participants completed questions regarding completion of four types of future planning documents: advance care directive, Enduring Guardian (someone appointed to make lifestyle and health decisions), a will, and Enduring Power of Attorney (someone appointed to make legal and financial decisions) by the person they support (yes, know, don’t know), including whether each of these documents were completed before or after receiving a dementia diagnosis. All participants were asked to indicate whether they or the person with dementia had discussed advance personal planning or planning ahead for the future following the dementia diagnosis with a general practitioner, geriatrician, nurse, lawyer/solicitor, family member, friend, other person or if no one had talked about it.

#### Timing

Participants were asked when they thought would be the best time for a health care provider to raise advance care planning following a dementia diagnosis. To ensure participants considered both the benefits and consequences of early and late discussions about advance care planning, the following information was first presented: “*Some people think discussions about advance care planning should happen at the time of a diagnosis of dementia*, *or soon after*, *so people have the chance to plan for their future care before they lose the capacity to do so*. *Others think discussions about advance care planning will be too distressing for people who have only recently been diagnosed with dementia*, *and that healthcare providers should wait to discuss this*. *When do you think would be the best time for a healthcare provider to initiate a conversation about advance care planning*?*”*. Response options included: at the time of receiving a diagnosis of dementia; in the first few weeks or months following a diagnosis of dementia; only when symptoms of dementia start getting worse; whenever the healthcare provider thinks it is appropriate; and healthcare providers should not discuss this at all.

#### Sociodemographic

Participants were asked to self-report their age, sex, marital status, education, employment, their relationship to person living with dementia they support, if they live with the person living with dementia they support, and if they have any chronic health conditions. They were also asked questions about the person with dementia who they support including their age, sex, the type of dementia they had been diagnosed with, the time since diagnosis, how long they had been providing care, and if they had any other chronic health conditions. Participants were also asked to rate the severity of dementia symptoms for the person they support using a ten-point Likert scale from 1 (very mild) to 10 (severe). Carers were also asked for their postcode which was used to calculate: rurality (city or regional) based on the Accessibility/Remoteness Index of Australia Plus (ARIA+) score; and the Socio-Economic Index for Areas (SEIFA), which provides an estimate of the degree of socio-economic advantage or disadvantage across geographical areas [27].

### Statistical analysis

Analyses were conducted in Stata version 15 [28]. Characteristics of participants and responses to advance care planning questions are reported as mean (standard deviation) for continuous variables and number (proportion) for categorical variables. As the proportion of missing data was low (<10%) a complete case approach was taken for each item. The association between having made an advance care directive and the factors of interest (age, sex, time since diagnosis, presence of other health conditions, symptom severity, SEIFA and ARIA+ score) of the person with dementia were modelled using univariable logistic regressions. All factors were then included in a single multivariable logistic regression. Estimates are expressed as Odds Ratios, modelling the odds of having an advance care directive. The assumption of linearity for the three continuous variables (age, symptom severity and SEIFA) was checked by plotting the log odds of the outcome against them.

### Ethics approval

This project received ethics approval from the Hunter New England Human Research Ethics Committee (17/05/17/4.07 and 18/07/18/4.06) and was registered with the University of Newcastle Human Research Ethics Committee (H-2018-0308). Completion and return of the survey was taken as implied consent to participate in the study.

## Results

### Sample

A total of 198 carers of people with dementia participated (48% consent rate). The sociodemographic characteristics of carers, and the people with dementia they support, are outlined in Table 1. Most participants were female (74%), were a partner (78%) or parent (19%) to the person with dementia they were caring for, and time since diagnosis of the person they cared for was less than 5 years (73%). The average age of carers was 70.6 years (SD = 11.4).

**Table 1:** Sociodemographic characteristics of carers, and the people with dementia they support (n = 198).

	Variable		N (%)
**Carer Characteristics**	**Age **	<60	35 (18%)
		60–69	33 (17%)
		70–79	86 (44%)
		≥ 80	42 (21%)
		Missing	2
	**Sex**	Male	50 (26%)
		Female	146 (74%)
		Missing	2
	**Marital status**	Married or partner	175 (89%)
		Single, divorced, separated, or widowed	22 (11%)
		Missing	1
	**Education**	High School or below	75 (42%)
		Vocational training, University or other	104 (58%)
		Missing	19
	**Employment**	Working: FT/PT/Casual	26 (13%)
		Not working	170 (87%)
		Missing	2
	**Living arrangement**	Lives with person they support	177 (91%)
		Lives separately to the person they support	18 (9%)
		Missing	3
	**Health conditions**	No chronic health conditions	58 (32%)
		At least one chronic condition	123 (68%)
		Missing	17
	**Relationship to person with dementia**	Partner	152 (78%)
		Parent	37 (19%)
		Other (in-law, grandparent, ex-partner)	8 (4%)
		Missing	1
**Characteristics of the person with dementia whom the carer supports**	**Age**	<60	3 (2%)
		60–69	18 (9%)
		70–79	67 (34%)
		≥ 80	107 (55%)
		Missing	3
	**Sex**	Male	114 (58%)
		Female	81 (41%)
		Missing	3
	**Type of dementia**	Alzheimer’s	106 (55%)
		Vascular dementia	19 (10%)
		More than one or other	30 (3%)
		Don’t know	36 (19%)
		Missing	7
	**Time since diagnosis**	less than 1 year	15 (8%)
		1–2 years	25 (14%)
		2–5 years	93 (52%)
		over 5 years	47 (26%)
		Missing	18
	**Time in caring role**	less than 1 year	12 (6.5%)
		1–2 years	22 (12%)
		2–5 years	97 (53%)
		over 5 years	53 (29%)
		Missing	14
	**Symptom severity rating**	≤ 3	20 (11%)
		4–6	101 (55%)
		≥ 7	63 (34%)
		Missing	14
	**Other health conditions**	No other conditions	49 (27%)
		At least one other condition	134 (74%)
		Missing	15

### Participation in future medical and financial planning

Table 2 shows which documents participants had prepared and when they were prepared. Almost all participants reported that the person with dementia they support had made a Will (97%), appointed an Enduring Guardian (93%), and appointed an Enduring Power of Attorney (89%). Only 47% had completed an advance care directive. Most documents were reported to have been completed before being diagnosed with dementia. Overall, 3 (1.5%) carers reported that the person with dementia they support had not completed any of the four documents asked about. In contrast, 45% (n = 89) of carers reported that the person with dementia they support had completed all 4 documents, 44% (n = 87) had completed 3, 5.1% (n = 10) had completed 2, and 4.6% (n = 9) had completed only one. There were no significant associations between the characteristics of the person with dementia, and whether they reported having prepared an advance care directive (Table 3).

**Table 2:** Number (%) of participants who have completed advanced care planning (N = 198).

	Done before the diagnosis of dementia	Done after the diagnosis of dementia	Total completing
**Made an Advance Care Directive**	54 (27%)	42 (21%)	96 (48%)
**Appointed an Enduring Guardian**	123 (62%)	52 (26%)	175 (88%)
**Made a Will**	170 (86%)	22 (11%)	192 (97%)
**Appointed an Enduring Power of Attorney**	135 (68%)	48 (24%)	183 (92%)

**Table 3:** Characteristics associated with having an advance care directive (N = 159).

Characteristic	Level of characteristic	Crude		Adjusted	
		Odds ratio (95% CI)	P value	Odds ratio (95% CI)	P value
**Sex of person with dementia**	Male versus Female	0.99	0.989	1.16	0.659
**Time since diagnosis**	< 2 years versus ≥2 years	1.75	0.122	1.51	0.344
**Rurality**	City versus regional	0.99	0.985	0.77	0.562
**Other health conditions**	No versus yes	1.2	0.573	1.27	0.529
**Age of person with dementia**		1.01	0.528	1.02	0.392
**Symptom severity**		1.06	0.481	1.06	0.554
**SEIFA**		0.99	0.186	0.99	0.097

### Discussion of advance care planning following a diagnosis of dementia

Table 4 reports the categories of people reported as having spoken about advance care planning with either the carer or person with dementia following diagnosis. Discussions about advance care planning had most often occurred with Geriatricians (53%, n = 103) and GPs (51%, n = 100). Nineteen percent of respondents (n = 38) indicated that no one had talked to them or the person they support about advance care planning following the dementia diagnosis.

**Table 4:** Who advance care planning was discussed with following a diagnosis of dementia (N = 196)*.

	N (%)
**Geriatrician**	103 (53%)
**General practitioner**	100 (51%)
**Family member**	79 (40%)
**Lawyer/solicitor**	60 (31%)
**Nurse**	48 (24%)
**Friend**	20 (10%)
**No one**	38 (19%)

* Participants could select more than one response so totals do not sum to 196. Row totals represent the total proportion of the whole sample selecting the response option.

### Preferences for timing of discussions about advance care planning

Most carers thought that discussions about advance care planning should occur in the first few weeks or months following a diagnosis of dementia (32%, n = 57), or when the healthcare provider thinks it is appropriate (31%, n = 55). A quarter of participants (25%, n = 45) thought discussions should occur at the time of diagnosis, and 12% (n = 21) thought that conversations should occur only when symptoms of dementia start getting worse. Only 1% (n = 1) thought that healthcare providers should not discuss advance care planning at all.

## Discussion

Overall, 88% of participants reported that the person living with dementia who they support had appointed an enduring guardian, and rates of financial planning were greater than 90%. These rates are consistent with other studies internationally [14, 20]. In contrast, carers reported that almost half of people with dementia did not have an advance care directive, despite being at substantial risk of future decisional incapacity as a result of their diagnosis [14, 20]. Other work has explored engagement in and preferences for advance care planning from the perspective of family caregivers caring for loved ones with young-onset dementia [29] and found similarly low levels of engagement in advance care planning, with plans for the future typically relating to non-medical affairs. Careful consideration should be given to the discrepancies in participation in financial planning compared to medical planning and understanding the reasons underpinning these differences. It is unclear what accounts for the disparate rates of completion of these differing advance care planning processes. It is possible that low rates of completion of advance care directives may be the result of uncertainties about the trajectory of deterioration of health, unwillingness or inability to anticipate potential future ill-health scenarios, the complex discussions and decisions involved in making future healthcare decisions, and/or a lack of understanding of the likely trajectory of disease, particularly in the case of dementia [24, 30].

Almost all carers of people with dementia believe advance care planning should be discussed by healthcare providers following a diagnosis of dementia. In this sample, advance care planning discussions with GPs and geriatricians occurred for approximately half of the sample, and advance care planning conversations had occurred with a lawyer/solicitor or family member for more than a third. Discussion of advance care planning with lawyers and solicitors may be a result of opportunistic discussions about advance care planning during financial planning. However, one in five had not had any discussions about advance care planning. Barriers and enablers to engaging in discussions about advance care planning have been well explored from both patient [31–33] and healthcare provider [16, 32, 34, 35] perspectives across settings, countries and population groups. There is strong evidence that positive perceptions about advance care planning and beliefs about its benefits do not necessarily translate into more end-of-life conversations [4]. Healthcare providers report lacking the time, resources, training and confidence to initiate and implement advance care planning [14]. Recent work has explored educational strategies to encourage discussion of advance care planning with people with dementia by general practitioners, finding that the inclusion of interactive and didactic components, targeting patients motivated and/or willing to participate in advance care planning, and the discussion of non-medical preferences as a starting point to discussions were key to effective advance care planning uptake [36, 37]. Other work has suggested that adequate knowledge and skills amongst patients, family and health-care professionals, patient and healthcare provider willingness to participate in advance care planning, strong relationships, effective administrative systems and contextual factors including embedding advance care planning into routine or standard care are important in residential aged care settings [38]. Effective models that promote discussion and documentation of advance care planning documents for people with dementia are needed.

There was variability in preferences for timing of discussion about advance care planning, however more than half of participants believed advance care planning discussions should occur at diagnosis or in the weeks/ months following a diagnosis. A further quarter of participants believed that healthcare providers should decide the appropriate time for discussion. Previous studies have highlighted the difficulties in understanding the right time to commence advance care planning discussions given the complexities of balancing the need to understand and accept the diagnosis of a serious illness, and having advance care planning documents in place before capacity is impacted [14, 19, 39, 40]. In interviews, people with dementia often expressed a reluctance to plan or consider too far into the future when considering advance care planning [14]. While patients’ readiness to engage in advance care planning is often considered a prerequisite for starting advance care planning conversations by healthcare professionals, uncertainty about a patient’s readiness to discuss advance care planning has been shown to impede the uptake of advance care planning in clinical practice [41, 42]. Our findings suggest that caregivers of patients with dementia are overwhelmingly supportive of healthcare providers initiating discussions early, or at the discretion of their healthcare provider. Further studies are needed to explore whether this finding aligns with the preferences expressed by people with dementia. Information could also be provided to caregivers soon after diagnosis about how they can initiate these conversations with the person they support and the type of questions they could consider together.

### Limitations

Study findings should be considered with regards to several limitations. Firstly, study participants were recruited using convenience methods and thus our sample is likely not reflective of all carers of people with dementia. Secondly, given that most participants were providing support to a person who had been diagnosed with dementia more than 2 years ago, it is also possible that recall bias and limitation in recall may affect data related to discussion of advance care planning following a diagnosis of dementia given the length of time between diagnosis and when data was collected. Thirdly, data were collected using a one-time cross-sectional survey and included only a small sample of carers with a modest overall consent rate. Finally, caregivers’ perceptions were examined rather than the perceptions of persons living with dementia due to difficulties accessing people living with dementia to participate Future research should obtain the perspectives of persons living with dementia and healthcare providers to contrast and contextualise these findings.

## Conclusions

Despite being at substantial risk of future decisional incapacity, more than half of people with dementia do not have an advance care directive. Carers of people with dementia believe advance care planning should be discussed by healthcare providers following a diagnosis of dementia.
